# Interictal magnetic signals in new‐onset Rolandic epilepsy may help with timing of treatment selection

**DOI:** 10.1002/epi4.12884

**Published:** 2024-01-04

**Authors:** Fengyuan Xu, Yihan Li, Yingfan Wang, Siyi Wang, Fangling Sun, Xiaoshan Wang

**Affiliations:** ^1^ Country Department of Neurology The Affiliated Brain Hospital of Nanjing Medical University Nanjing China

**Keywords:** BECTS, CECTS, MEG, SeLECTS

## Abstract

**Objective:**

With research progress on Rolandic epilepsy (RE), its “benign” nature has been phased out. Clinicians are exhibiting an increasing tendency toward a more assertive treatment approach for RE. Nonetheless, in clinical practice, delayed treatment remains common because of the “self‐limiting” nature of RE. Therefore, this study aimed to identify an imaging marker to aid treatment decisions and select a more appropriate time for initiating therapy for RE.

**Methods:**

We followed up with children newly diagnosed with RE, classified them into medicated and non‐medicated groups according to the follow‐up results, and compared them with matched healthy controls. Before beginning follow‐up visits, interictal magnetic data were collected using magnetoencephalography in treatment‐naïve recently diagnosed patients. The spectral power of the whole brain during initial diagnosis was determined using minimum normative estimation combined with the Welch technique.

**Results:**

A difference was observed in the magnetic source intensity within the left caudal anterior cingulate and precentral and postcentral gyri in the delta band between the medicated and non‐medicated groups. The results revealed good discriminatory ability within the receiver operator characteristic curve. In the medicated group, there was a specific change in the frontotemporal magnetic source intensity, which shifted from high to low frequencies, compared with the healthy control group.

**Significance:**

The intensity of the precentral gyrus magnetic source within the delta band showed good specificity. Considering the rigor of initial treatment, the intensity of the precentral gyrus magnetic source can provide some help as an imaging marker for initial RE treatment, particularly for the timing of treatment initiation.

**Key points**

- The study aimed to identify imaging markers to help with Rolandic epilepsy (RE) treatment.
- Children with initial RE were grouped as per the post‐outcome follow‐up.
- MEG data during initial diagnosis were a predictor after follow‐up grouping.
- The identified marker may help with the timing of medication in children with RE.

## 1 INTRODUCTION

Rolandic epilepsy (RE), also called benign epilepsy with central temporal spikes (BECTS), is one of the most common epilepsy syndromes during childhood with a significant age dependence.^1^ The seizures often start in middle childhood, between 4 and 10 years old, ending during puberty.^2^ Predictably, the seizures occur during sleep, frequently in the early morning, and occasionally occurring throughout the day.^2^ Therefore, RE is a “benign” disease with a good prognosis. Antiseizure medication (ASM) for RE has been controversial due to the benign course of RE and drug side effects.

Numerous studies have examined how RE affects behavioral and neuropsychological functioning. Some children with RE are more likely to exhibit cognitive deficits, behavioral disorders, learning disabilities, specific language disorders, attention deficits, and educational and academic issues.^3^ Additionally, some patients with RE exhibit atypical symptoms.^4^ Examples include aberrant electroencephalogram (EEG) characteristics that differ from typical RE and the persistent failure of traditional ASM therapy. The International League Against Epilepsy has altered the name of “BECTS” to “Self‐limited epilepsy with centrotemporal spikes (SeLECTS)” and does not advise using the term “benign”.^5^

Many researchers advocate pharmacological therapy because some RE patients are not benign.^2^, ^6^ However, most experts believe that treatment should be delayed until patients experience the second or third seizure.^2^ Most children with RE have two to five seizures,^7^ and some have only one seizure in their lifetime. Therefore, this group of children with only one attack does not need medication. There are two frequently asked questions by parents when they observe a child with RE suffering from their first seizure. The first question is whether the child will experience another seizure, and the second is whether any medication is required. However, these questions cannot be answered reliably for children with RE after their first seizure. Based on clinical experience, parents can be advised to only observe and defer medication use until their child has experienced their second or third seizure. However, delay may hold treatment in children with a poorer prognosis in the future. Moreover, such measures may increase worry and anxiety in both the patient and their parents. The presence of discernible markers can assist medical professionals in deciding treatment initiation in patients. In addition, such markers can help decide between commencing therapy and deferring it, enabling optimal support to patients. Therefore, we sought markers for guidance in the early stages of treatment.

Imaging indicators have been essential among the numerous epilepsy markers as they are non‐invasive.^8^ Numerous investigators have also searched for imaging markers that predict the prognostic relevance of RE development.^9^, ^10^ However, the need for treatment in primary RE has not been expected. This can also be somewhat predictive of the frequency of RE episodes due to the absolute clinical correlation between treatment for RE and the number of seizures.^11^, ^12^ With the rapid advancement of imaging technology and electrophysiological tests, more non‐invasive examinations of the brain, including functional magnetic resonance imaging (fMRI), EEG, and others, have been employed for brain imaging.^13^, ^14^ Magnetoencephalography (MEG) is a new detection technique possessing excellent temporal and spatial resolution. The MEG signal can flow through the cerebral fluid, skull, and other subcutaneous tissues without attenuation, leading to low artifact interference.^15^

In this study, we followed up with treatment‐naïve children who had their first seizure. They were classified into medicated and non‐medicated groups based on clinical outcomes. We retrospectively analyzed their interictal MEG data without medication during initial diagnosis at the level of neuromagnetic source activity at different frequency bands inside the whole brain to provide therapeutic recommendations during the early stages of RE.

## 2 MATERIALS AND METHODS

### 2.1 Participants

Fifty‐eight children with RE aged 6–13 years were diagnosed and recruited between September 2017 and June 2020 from the Department of Neurology, Nanjing Brain Hospital, and Nanjing Children's Hospital. The included children were not on ASMs and satisfied the International League Against Epilepsy (ILAE) 2017 seizure classification criteria^16^ at the first diagnosis. Extensive clinical information, including age at first seizure, frequency, and duration of seizures, as well as medical and family history, were collected during patient enrollment. At 30–63 months after MEG recording (MEG data recording in RE children who were initially diagnosed and not on any intervening medication), we assessed all patients at follow‐up and collected relevant information regarding the use of ASMs and seizures. Finally, 44 patients with RE were included in this study as 10 were lost to follow‐up, and 4 did not meet the inclusion criteria. After follow‐up, the children were classified into two groups based on the outcome of whether the patients were prescribed ASMs (ASM criteria: children with two or more episodes within 3 months^2^). Twenty‐seven children (female/male: 15/12, average age: 8.20 ± 1.25 years) were included in the ASM group and 17 (female/male: 9/8, average age: 8.41 ± 1.81 years) in the group not taking ASMs.

The inclusion criteria for patients with RE were as follows: (i) newly diagnosed with RE according to the diagnostic criteria^16^; (ii) an uncomplicated pregnancy and delivery, normal neonatal health, early psychomotor development, and regular school enrolment; and (iii) no other neurological, psychiatric, or somatic problems or aphasia. The exclusion criteria were as follows: (i) evidence of symptomatic epilepsy from brain MRI and medical history; and (ii) inability to comply with or comprehend the research protocols. Notably, all the children with RE had been seizure‐free for at least 3 days before data collection.

An additional 27 typically developing children (female/male: 16/11, average age: 8.37 ± 1.94 years) without any history of neurological, psychiatric, or other major medical issues were recruited as healthy controls (HCs). They were matched to the RE children according to age, sex, grade, residential area (urban/rural), and socioeconomic status. The experimental protocol was explained to all the participants and their parents. The parents signed the written informed consent approved by the Ethics Committee of Nanjing Brain Hospital.

### 2.2 Neuropsychological assessments

The Chinese version of the Fourth Edition of the Wechsler Intelligence Scale for Children (WISC‐IV) was used to investigate the intelligence level of all the included patients with RE.^17^ The scale has 10 core subtests with five additional subtests. The score of each subtest is obtained using standard norm conversion, making the subject scores comparable for different ages. The full‐scale intelligence quotient (FSIQ) was used to measure cognitive ability, as it showed good reliability in previous studies.^18^ All tests for the WISC‐IV were administered by clinicians specializing in child neuropsychology, and all neuropsychological assessments were performed on the same day as the MEG data recording during the initial RE diagnosis.

### 2.3 MEG recordings

The initial imaging was conducted in a magnetic‐shielded room at the MEG Center of Nanjing Brain Hospital using a whole‐head, 275‐channel MEG system (VSM MedTech Systems, Inc., Coquitlam, BC, Canada). Three electromagnetic coils were affixed to the reference landmarks on the left and right pre‐auricular points and the nasion of each participant before MEG recording to determine the head position. During scanning, the patients were encouraged to close their eyes, remain silent, be awake and calm, and clear their minds. We recorded at least four MEG data files for each patient for 2 min at a sample rate of 6000 Hz. We communicated with the patients through a microphone to ensure that they were awake before collecting data. If a sleep fragment appeared, the data were deleted and re‐recorded. The head position of each subject was measured at the beginning and end of each scan to limit the head mobility to <5 mm.

All patients were scanned on a 3T MRI scanner (Siemens, Munich, Germany). Anatomic 3D T1‐weighted images were procured with a rapid gradient echo sequence (TR/TE = 1900/2.48 ms). The following imaging parameters were involved: the field of view was 250 × 250 mm^2^; the flip angle was 9°; the voxel size was 0.48 × 0.48 × 1 mm^3^; and the matrix was 512 × 512. For each subject, 176 sagittal slices were collected, and a foam cushion was used to enhance comfort and reduce motion. Three markers were affixed in the nasion and pre‐auricular regions of each patient to minimize the MRI and MEG data matching bias. Moreover, the relative location of the head of each patient was quantified relative to the MEG sensor.

### 2.4 Data pre‐processing

The following strategies helped eliminate non‐brain and environmental artifacts from spontaneous MEG data. (i) All data were visually reviewed for segments having artifacts induced by head movements or external noise while deleting the contaminated segments. (ii) Notch filters (50 Hz and harmonics) were applied to decrease powerline contaminations. (iii) The MEG recordings were started with a 2 min empty room recording to obtain ambient and sensor noise. The noise covariance was determined using offline source analysis to account for residual and stationary instrumental, sensor, and environmental noise components. In addition, T1‐weighted structural volume images were automatically reconstructed on the surface model for source investigation with a FreeSurfer image analysis package.* We generated the topographical 3D descriptions of the brain surface using integrated geometric reconstructions of the scalp. Brain gray and white matter were utilized to estimate the boundaries.

### 2.5 Spectral power

We used depth‐weighted minimum–norm estimations (MNE) to compensate for the susceptibility of MEG to radial source components. The current strength dynamics of cortical sources were determined from EEG data with depth‐weighted MNE.^19^ This method can detect concurrent current sources distributed across the entire cortical surface.^19^ Furthermore, it can outperform MNE without depth weighting regarding spatial accuracy.^20^, ^21^ A forward solution was observed by employing a multiple overlapping spheres model. This shows each cortical vertex as a current dipole and includes approximately 15 000 vertices and depth‐weighted MNE for estimating the scattered source model from the MEG data.^19^, ^22^, ^23^ The inverse operator helped estimate the current source distribution as follows. (i) The source orientations had been constrained to be normal to the cortex surface. (ii) The depth weighting algorithm was used to compensate for the inhomogeneous sensitivity of the current flow with the depth and orientation.^21^ The current study utilized the dSPM option to standardize the data concerning global noise and data covariance statistics. The resulting dSPM maps were a set of *z*‐scores. (iii) A regularization value *λ* ^2^ = 0.33 decreased numerical instability, lowered the noise sensitivity of MNE, and created a spatially smoothed solution.^19^ It was defined as the reciprocal of the signal‐to‐noise ratio (SNR) of MEG recordings. The default SNR in the Brainstorm software is “3,” adopting the original SNR notion in the MNE software.^23^ Brainstorm^24^ is a published software under the GNU universal general public license. It was used to perform the depth‐weighted MNE study.

This study did not evaluate specific regions of interest (ROIs). Instead, the entire brain was examined using Desikan–Killiany cortical parcellation. The relative current power of all vertices in the ROI was considered to estimate the oscillatory power based on the source. The Welch technique (window duration 5 s with 50% overlap) was used to determine the power spectral density (PSD) of each ROI.^25^, ^26^ According to our previous studies,^27^, ^28^ a 30s interictal waveform was selected for each subject, eliminating spikes as much as feasible. The waveform segments were examined in the five frequency bands: delta (2–4 Hz), theta (5–7 Hz), alpha (8–12 Hz), beta (15–29 Hz), and gamma (30–59 Hz).

We scaled the PSD values proportionally to the total power over the whole frequency spectrum at each frequency bin: Relative $\mathrm{PSD}\left(f\right)=\mathrm{PSD}\left(f\right)/\sum_{i}\left[\text{Total}\;\mathrm{PSD}\left(f_{i}\right)\right]$, where $f_{i}$ indicates the individual frequency from the absolute PSD. The PSD readings were standardized across brain areas and participants utilizing this approach.^25^

### 2.6 Statistical analysis

We compared the demographic and clinical data between the ASM and non‐ASM groups using an independent samples *t*‐test or a chi‐squared test. The data distribution was assessed using the Kolmogorov–Smirnov test. Then, the Kruskal–Wallis test was applied to evaluate group differences in the neuropsychological assessment and spectral power for each ROI within each frequency band for the three subject groups. We utilized *p* < 0.05 as the threshold for statistical significance. It was corrected using false discovery rate (FDR) based on multiple‐comparison corrections. Specifically, at the regional level (spectral power), there were 68 ROIs (whole brain), 5 frequency bands, and 3 groups of subjects. Therefore, the *p*‐value in the spectral power analysis had to be corrected 68 × 5 × 3 times. Furthermore, Kruskal–Wallis tests demonstrated differences between the three groups, and spectral power with significant differences was calculated between the ASM and non‐ASM groups. The diagnostic value of the ASM group was determined using receiver operator characteristic (ROC) curve analysis. Moreover, the area under the curve (AUC) was plotted to determine diagnostic performance. All the data are represented as mean and standard deviation (SD). SPSS 24.0 was used for the statistical analyses (SPSS Inc., Chicago, IL, USA).

## 3 RESULTS

### 3.1 Subjects

The current study comprised 44 individuals with RE and 27 HCs. The patient population included 27 ASM users and 17 patients with RE who did not use ASMs. The clinical information and the statistical analyses for the two groups during enrollment are demonstrated in Table 1. Children in the HC group had comparable age and gender ratios with the other two groups. No significant differences were observed between the ASM and non‐ASM groups and between the non‐ASM and HC groups in the neuropsychological assessment, as depicted in Table 1. After 30–63 months of follow‐up, 12/17 children without medication had one seizure. By contrast, 5/17 had two, and 2/17 children experienced daytime seizures, whereas the rest had nocturnal seizures. Of the 27 children on ASMs, 17 were on levetiracetam, 6 used oxcarbazepine, and 4 were on valproic acid. Most children 21/27 in the ASM group suffered seizures only at night or before waking up in the morning. Eighteen children were effectively managed on ASMs without repeated seizures, and six had one to two episodes after taking medicine. Additionally, three had poor drug responses and many seizures after taking the medication.

**TABLE 1:** Clinical data of the children, mean ± SD.

Clinical characteristics	ASMs group (*n* = 27)	Non‐ASMs group (*n* = 17)	Healthy controls (*n* = 27)	*p*: ASMs vs. non‐ASMs	*p*: ASMs vs. HCs	Non‐ASMs vs. HCs
Sex						
Female	15	9	16	0.865	0.680	0.783
Male	12	8	11			
Age at seizure onset, years	8.14 ± 1.28	8.34 ± 1.81		0.70		
Age at scan, years	8.20 ± 1.25	8.41 ± 1.81	8.37 ± 1.94	0.68		
Epilepsy course, months	0.88 ± 0.95	0.99 ± 1.20		0.73		
Number of seizures	1.37 ± 0.49	1.29 ± 0.47		0.61		
FSIQ	90.04 ± 10.89	97.00 ± 10.16	104.59 ± 10.38	0.487	0.0002*	0.070

*Note*: Clinical information of the patients.

Abbreviations: ASM, antiseizure medications; FSIQ, Full‐scale Intelligence Quotient; HCs, healthy controls.

* The *p*‐values were <0.05.

### 3.2 Spectral power

The results of the three groups were analyzed using the Kolmogorov–Smirnov test, which indicated statistical differences in the delta‐, alpha‐, beta‐, and gamma‐frequency bands within some brain regions. The results of the specific brain regions and comparisons among the groups have been described below.

#### 3.2.1 ASM and non‐ASM group

**FIGURE 1:**
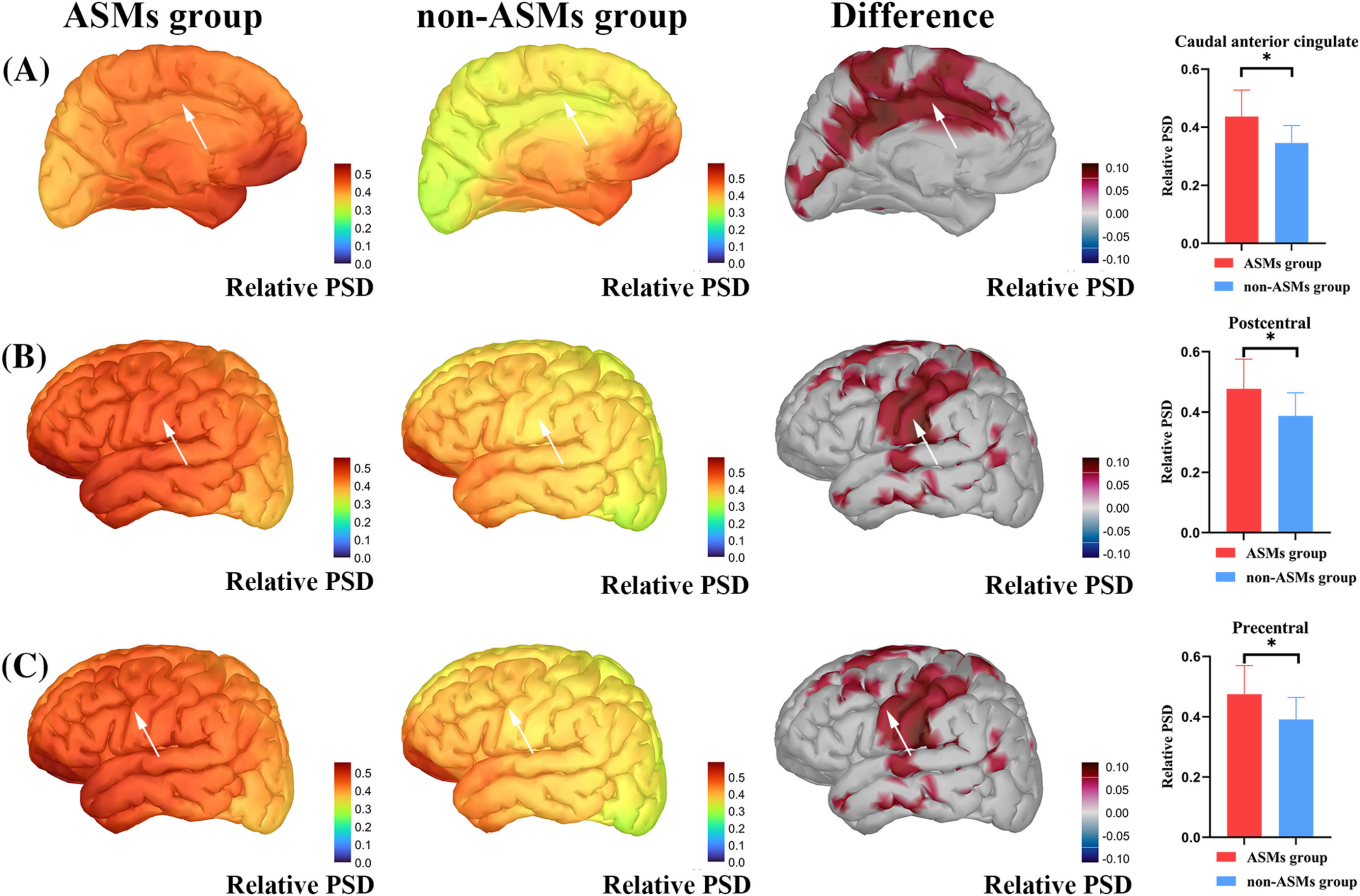
Relative power spectral density (PSD) activation maps in the delta band between the antiseizure medication (ASM) and non‐ASM groups in A (left caudal anterior cingulate gyrus), B (left postcentral gyrus), and C (left precentral gyrus). The figure also includes maps of the differences (between ASM and non‐ASM groups) and bar graphs depicting specific differences. Arrows in the plots show the positions of the caudal anterior cingulate gyrus, postcentral gyrus, and precentral gyrus. Colors represent activation maps to represent activation intensity. The difference map activation threshold was set to 70%. The bar graphs demonstrate the mean ± standard deviation of the relative PSD. *After correction, the *p*‐values were <0.05.

The two groups had statistical differences in some brain regions in the delta band after analyzing and correcting the magnetic source intensity of the whole brain (68 ROIs) within the delta, theta, alpha, beta, and gamma bands. The magnetic source intensity of the ASM group was higher than that of the non‐ASM group in the left caudal anterior cingulate (*p* = 0.001, *p = 0.02), precentral gyrus (*p* = 0.003, *p = 0.04), and postcentral gyrus (*p* = 0.003, *p = 0.04) (*p: after FDR correction). The specific magnetic source strengths and differences between the two groups are represented in Figure 1.

#### 3.2.2 Non‐ASM and healthy control groups

No statistically significant differences were observed in the intensity of the total brain magnetic sources among the five frequency bands between these two groups.

#### 3.2.3 ASM and healthy control groups

**FIGURE 2:**
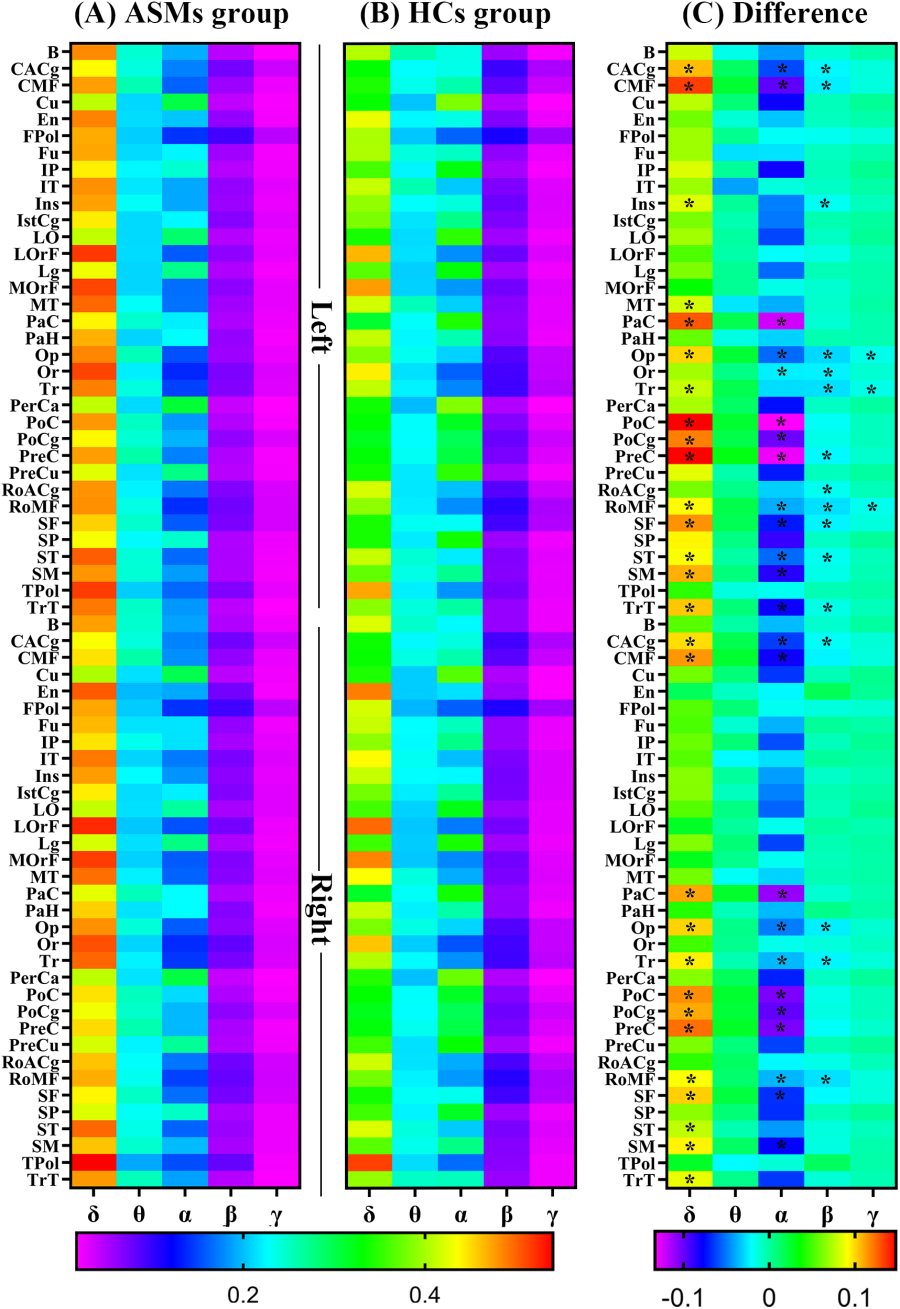
Relative power spectral density (PSD) activation heatmaps for the ASM (A) and healthy control groups (B) within the five frequency bands of the whole brain (68 ROIs) and the difference maps (C) between the two groups (A, B). (A, B) Activation maps share a color scale, depicting the activation intensity. The different map colors characterize the size difference between A and B. The full range of brain regions has been demonstrated in Table 2.

Statistical differences could be identified in various brain regions in the delta‐, alpha‐, beta‐, and gamma‐frequency bands between the two groups. There were many variances within the frequency bands of the same brain regions. Figure 2 demonstrates the differences between ASM and the healthy control groups in each frequency band. *p: *p*‐value after FDR correction. The abbreviation details in the figure are depicted in Table S1. In addition, owing to the large number of results for the two groups, non‐significant outcomes have not been listed in detail for clarity. The results that have been corrected for significance are described in Table 2. All statistical results can be found in Data S1.

**TABLE 2:** Brain areas showing the significant magnetic source intensity between the ASM and healthy control groups.

Frequency band	Left brain regions	*p*‐Value	*p*‐Value*	Right brain regions	*p*‐Value	*p*‐Value*
Delta	Caudal anterior cingulate	4.00E‐05	0.002	Caudal anterior cingulate	0.004	0.01
	Caudal middle frontal	1.00E‐06	0.0003	Caudal middle frontal	2.00E‐05	0.002
	Insula	0.001	0.02	–	–	–
	Middle temporal	0.002	0.03	–	–	–
	Paracentral	6.70E‐05	0.003	Paracentral	0.0004	0.01
	Pars opercularis	4.00E‐05	0.002	Pars opercularis	2.50E‐05	0.002
	Pars triangularis	0.001	0.02	Pars triangularis	0.0003	0.01
	Postcentral	1.00E‐06	0.0003	Postcentral	3.10E‐05	0.002
	Posterior cingulate	1.10E‐05	0.001	Posterior cingulate	5.80E‐05	0.003
	Precentral	3.82E‐07	0.0003	Precentral	3.00E‐06	0.0004
	Rostral middle frontal	0.0004	0.01	Rostral middle frontal	0.001	0.02
	Superior frontal	8.00E‐06	0.0008	Superior frontal	9.90E‐05	0.004
	Superior temporal	0.0004	0.01	Superior temporal	0.0004	0.01
	Supra marginal	6.20E‐05	0.003	Supra marginal	0.001	0.004
	Transverse temporal	0.0002	0.006	Transverse temporal	0.0004	0.01
Alpha	Caudal anterior cingulate	0.002	0.03	Caudal anterior cingulate	0.001	0.02
	Caudal middle frontal	2.00E‐06	0.0004	Caudal middle frontal	8.30E‐05	0.004
	Paracentral	0.0005	0.01	Paracentral	0.001	0.02
	Pars opercularis	8.30E‐05	0.003	Pars opercularis	0.0004	0.01
	Pars orbitalis	0.003	0.04	–	–	–
	–	–	–	Pars triangularis	0.002	0.03
	Postcentral	3.00E‐06	0.0004	Postcentral	0.0004	0.01
	Posterior cingulate	0.0002	0.006	Posterior cingulate	0.0003	0.01
	Precentral	1.00E‐06	0.0003	Precentral	8.90E‐05	0.004
	Rostral middle frontal	4.00E‐06	0.0005	Rostral middle frontal	3.20E‐05	0.002
	Superior frontal	3.00E‐06	0.0004	Superior frontal	1.40E‐05	0.001
	Superior temporal	0.0008	0.02	–	–	–
	Supra marginal	0.0003	0.01	Supra marginal	0.0006	0.01
	Transverse temporal	0.0004	0.01	–	–	–
Beta	Caudal anterior cingulate	0.0009	0.02	Caudal anterior cingulate	0.003	0.04
	Caudal middle frontal	0.003	0.04	–	–	–
	Insula	0.001	0.02	–	–	–
	Pars opercularis	0.0001	0.004	Pars opercularis	0.0005	0.01
	Pars orbitalis	0.002	0.03	–	–	–
	Pars triangularis	0.0007	0.02	Pars triangularis	0.002	0.03
	Precentral gyrus	0.003	0.04	–	–	–
	Rostral anterior cingulate	0.001	0.02	–	–	–
	Rostral middle frontal	0.001	0.02	Rostral middle frontal	0.001	0.02
	Superior frontal	0.003	0.04	–	–	–
	Superior temporal	0.001	0.02	–	–	–
	Transverse temporal	0.002	0.04	–	–	–
Gamma	Pars opercularis	0.002	0.03	–	–	–
	Pars triangularis	0.002	0.04	–	–	–
	Rostral middle frontal	0.003	0.04	–	–	–

Abbreviations: ASMs, antiseizure medications; *p*‐values, uncorrected *p*‐value.

* *p*‐Value after utilizing the FDR for multiple comparisons (five frequency bands, 68 brain regions, and three groups).

### 3.3 Predictive value: ROC curves

**FIGURE 3:**
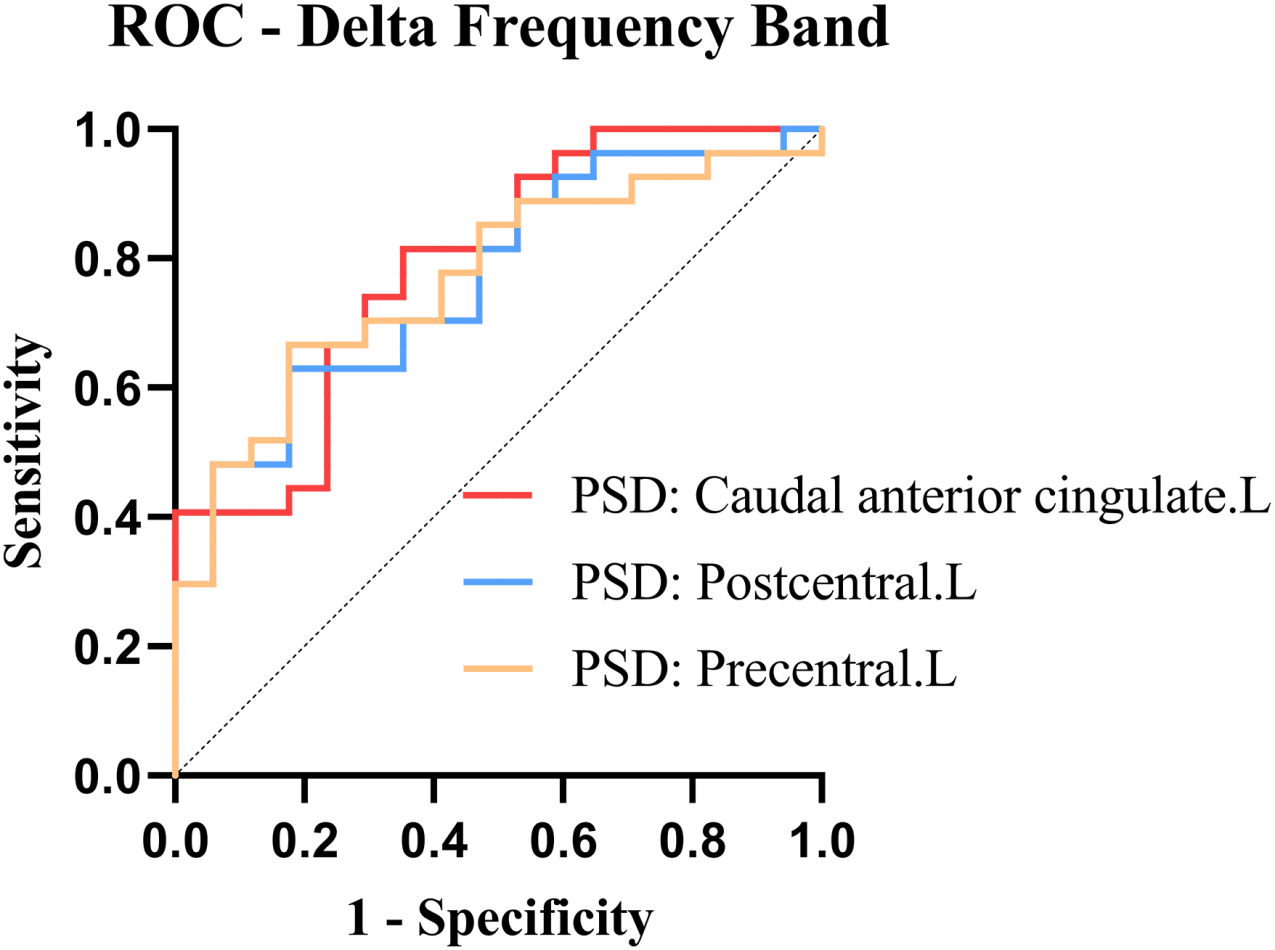
Receiver–operator characteristic (ROC) curves of the relative power spectral density (PSD) of the left anterior cingulate caudal gyrus, left central posterior gyrus, and left central anterior gyrus within the alpha band. It discriminates ASM RE from non‐ASM RE individuals.

The ROC curve helped find the predictors of using ASMs in early patients with RE after the Kruskal–Wallis test, indicating differences between the ASM and non‐ASM groups. The ROC curve revealed that the spectral power of the left caudal anterior cingulate [95% CI (0.652, 0.925); *p* = 0.001], left postcentral [95% CI (0.624, 0.905); *p* = 0.003], and left precentral [95% CI (0.630, 0.908); *p* = 0.003] were statistically significant in the alpha band. Figure 3 demonstrates the ROC curves for the three indicators. The specific AUC area, sensitivity, and specificity of the three indicators are listed as follows:

Left caudal anterior cingulate: AUC: 0.789, Sensitivity: 0.815, and Specificity: 0.647.
Left postcentral: AUC: 0.765, Sensitivity: 0.630, and Specificity: 0.824.
Left precentral: AUC: 0.769, Sensitivity: 0.667, and Specificity: 0.824.

## 4 DISCUSSION

The question of when to begin RE treatment has always been challenging. The opinion of the treating physician is usually decisive while advising parents on treatment.^29^ A rising number of researchers now advocate the use of medicines. However, most researchers consider additional indications for starting treatment, including early onset, multiple seizures at the onset, and higher seizure number (particularly generalized tonic–clonic seizures).^6^ Once ASMs are started, the adverse effects of the medication and the financial hardship of consuming them for an extended time period must be considered. Thus, we evaluated imaging markers for predicting the prognosis of RE treatment early to guide clinical treatment. The whole‐brain magnetic source spectrum energy activity levels were used and grouped according to follow‐up treatment outcomes. We may be more confident recommending medication if a child with RE has diagnostic criteria matching the imaging indicators. When it is anticipated that a child with RE could have a poor prognosis, the child can be treated as early as possible to avoid delays. However, if the imaging reveals a favorable prognosis, the child and parents can choose to defer treatment. To our knowledge, this is the first study using resting‐state MEG to investigate follow‐up therapy outcomes.

In the absence of clear guidelines for RE treatment, an individualized risk–benefit assessment is needed to make an informed decision about appropriate therapy. Many clinicians have attempted to categorize children with RE who are at high risk for poor prognosis using diverse measurements. For instance, children with “atypical” RE experience seizures more commonly, have atypical seizure types, and respond poorly to medications.^4^, ^30^ Van Klink et al.^31^ attempted to predict the frequency of seizures using the number of ripples detected in EEG. Their study indicated that more ripples could predict more frequent seizures.^31^ Furthermore, the spike–wave index (SWI) of the non‐rapid eye movement (NREM) phase has been shown to be clinically diagnostic and prognostic.^4^ Indeed, Tovia et al.^32^ suggested that an SWI of ≥85%, which consists mainly of cognitive deficits, frequent seizures, and poor response to medication, is a significant risk factor of poor prognosis in patients with RE. In addition, although the age of onset did not differ between the two groups in the present study (possibly owing to the small sample size), the correlation between the age of onset and RE prognosis has been most commonly reported, with a younger age of onset potentially suggesting a worse prognosis.^4^, ^30^, ^33^ Thus, examining children with poor RE prognosis is of utmost clinical importance. However, our follow‐up findings demonstrated that there were fewer patients in this group, which is characterized by frequent attacks and inadequate medication response, and more treatment‐responsive patients with two to five seizures. It is likely that within clinical settings, there is a higher proportion of children who experience two to five attacks, respond well to medication, and require therapeutic intervention. Thus, it is expected that the present study will benefit more children who are in need of treatment and not limited to atypical RE. Indeed, the patient's family decides whether and when to start treatment. The role of the physician is to inform, advise, and guide. However, additional research on more comprehensive prognostic indicators can arm physicians to better guide their patients.

In the search for markers, the difference in whole magnetic brain source intensity in different frequency bands between the ASM and non‐ASM RE patient groups was in some brain regions within the delta band. Enhanced activity in the delta band of the brain is believed to be a brain dysfunction.^34^ Thus, the improved magnetic source activity of the delta band in the left caudal anterior cingulate gyrus, precentral gyrus, and postcentral gyrus of the ASM group could represent abnormal brain activity. Thus, abnormal activity in these brain regions may be associated with RE‐related deficits. The origin sites of RE episodes, precentral gyrus and postcentral gyrus, are associated with the clinical symptoms of RE.^35^ Li et al.^36^ reported that children with RE had significantly increased connectivity in the sensorimotor network (SMN). Compared with HCs, this included the precentral and postcentral gyrus, which may lead to typical symptoms such as paresthesia and twitching of the mouth, face, and hands. Furthermore, aberrant functional integration has been identified between the SMN and language networks, which could be associated with language deficits in children with RE.^37^, ^38^ Research has indicated that seizure activity could be shifted from the sensorimotor cortex to other areas.^39^ Moreover, interictal epileptiform discharges may affect their function by spreading the corresponding functional networks. This could cause, for example, cognitive impairment and attention deficits.^36^ The functional connectivity of the dorsal attention network (DAN) was also significantly altered in children with RE.^36^ The anterior cingulate cortex (ACC) is a core area for integrating input from different sources to regulate cognitive processes, guide behavior,^40^ and maintain attention.^41^ Studies associated with childhood epilepsy identified that ACC plays an essential role in the attentional salience network (SN) and DAN. Moreover, the abnormal activity of ACC was associated with attentional deficits,^41^, ^42^ consistent with our study. Abnormal differences in the brain regions were present between the ASM and non‐ASM groups in this study. It was hypothesized that the ASM group is more affected by epilepsy and potentially performs worse in cognition and related attention. This could explain the need for medication in the ASM group. Our neuropsychological ratings did not show statistical differences between the two groups. However, the ASM group had lower cognitive scores on the borderline of normal cognition (90–110 points), and RE‐related cognitive deficits progressed with the disease.^43^ Due to sample size limitations, further confirmations are required.

When searching for a new imaging marker, the sensitivity and specificity must be balanced. Overly sensitive testing can lead to many children with RE requiring medication, whereas extremely stringent criteria may result in the underdiagnosis of those requiring medication. Most physicians prefer to extend the duration of treatment using ASMs^44^, ^45^ until 1–2 years after the previous seizure.^46^ Untreated or hasty drug discontinuation may cause seizures.^47^ However, research suggests that continuous anti‐epileptic drug exposure is not benign, as the most commonly used pharmaceuticals lead to attention impairments, aggression, anger, anxiousness, and tiredness among 30%–70% of patients.^48^, ^49^, ^50^, ^51^, ^52^ Studies have also indicated that medication alone can lead to cognitive impairment.^53^ Unwanted cognitive side effects are troubling due to the growing recognition of minor but prevalent cognitive impairments in RE.^3^ Therefore, sacrificing some sensitivity can boost specificity to avoid recommending the medicine to too many children with an early episode. This treatment strategy can be considered following the second episode in patients with RE with a missed diagnosis who require medication. Finally, we intended to provide clinicians with a diagnostic signal to propose no‐treatment alternatives with greater certainty in some patients. The overall AUC of the three imaging markers did not differ significantly in our study. With better sensitivity but somewhat lower specificity, the magnetic source intensity in the delta band caudal anterior cingulate gyrus showed the highest prediction accuracy. The magnetic source intensity of the precentral gyrus in the delta band had a greater specificity despite a somewhat lower sensitivity. Its AUC also demonstrated good prediction accuracy. Therefore, the magnetic source intensity of precentral gyrus in the delta band can be expected to be used as an imaging marker to guide the treatment of children with early RE. However, further diagnostic effects should be validated.

The healthy control group was added as a baseline. As the main objective was to investigate treatment‐related markers in the ASM and non‐ASM groups, only a brief overview of the results obtained from the healthy control group along with a comparison between the two groups was provided. Additional pathophysiological studies will be conducted in the future to specifically focus on these results. No significant changes were observed in magnetic source activity between the healthy control and non‐ASM groups. However, the ASM and healthy control groups had disparities in the magnetic source strength across numerous frequency bands. Therefore, the non‐ASM group is in a transitional phase between the healthy control and the ASM groups. In the early stages of the disease, the functional activation of the brain of RE children has not experienced significant flocculation. The ASM group had much stronger magnetic source activity in the lower delta bands and significantly reduced activity in the higher bands, such as alpha, beta, and gamma, compared with the healthy control and ASM groups. Therefore, the magnetic source activity of ASMs shifted from high‐frequency to low‐frequency activity. This spectral feature (especially the increased delta activity) corresponds to brain activity abnormalities.^34^ This spectral feature may be attributed to the impact of the RE disease itself.

It is important to note that this study has certain limitations. The sample size of this study was modest and should be increased in the future to validate the current results due to the limits of the loss to follow‐up. In addition, as certain patient data, such as seizure type, SWI, and other parameters, were not recorded at the time of enrolment, they were excluded from the study. Furthermore, no current worldwide MEG approach can ensure the total absence of artifacts and noise despite significant attempts at reducing artifacts and noise in the MEG signal. A larger sample size will be required to confirm the findings of this fact. Finally, the clinical diagnostic use of MEG may be restricted. Our study will facilitate relevant researchers in their search for RE treatment‐related imaging markers. Additionally, more popular methods, such as EEG‐ or MRI‐related studies, will corroborate our findings and provide clinical relevance.

In conclusion, our study revealed that a higher magnetic source intensity in the delta band of the whole brain is characteristic of children with early RE who require medication. The ROC curve analysis indicated that the magnetic source intensity in the central precentral gyrus of the delta band has good accuracy and specificity as an imaging marker to assist in selecting an optimal treatment initiation time.

## AUTHOR CONTRIBUTIONS

FX and YL conceptualized the entire study. FX, YL, SW, and FS extracted the raw data. Data analysis was performed by FX and YW. FX drafted the manuscript, and XW revised the draft. All the authors read and approved the final manuscript version.

## FUNDING INFORMATION

The current study was supported by the General Program of Natural Science Foundation of Jiangsu Province (Grant No. BK20191127), the Health Department of Jiangsu Province (Grant No. H2018062), the Medical and Health International Cooperation Project of Nanjing Municipal Science and Technology Bureau (Grant No. 201911044), and the National Natural Science Foundation of China (Grant No. 82071455).

## CONFLICT OF INTEREST STATEMENT

None of the authors has any conflict of interest to disclose.

## ETHICS STATEMENT

We confirm that we have read the Journal's position on issues involved in ethical publication and affirm that this report is consistent with those guidelines.

## Supporting information


Data S1:

Table S1.

## Data Availability

The datasets generated and analyzed during the current study are available from the corresponding author upon reasonable request.

## Footnotes

*
https://surfer.nmr.mgh.harvard.edu/.
