# Supplementary figures and images for: Interictal magnetic signals in new‐onset Rolandic epilepsy may help with timing of treatment selection
Source: Epilepsia Open. 2024 Jan 4;9(1):368–79. doi: 10.1002/epi4.12884 (PMC10839299; doi:10.1002/epi4.12884)

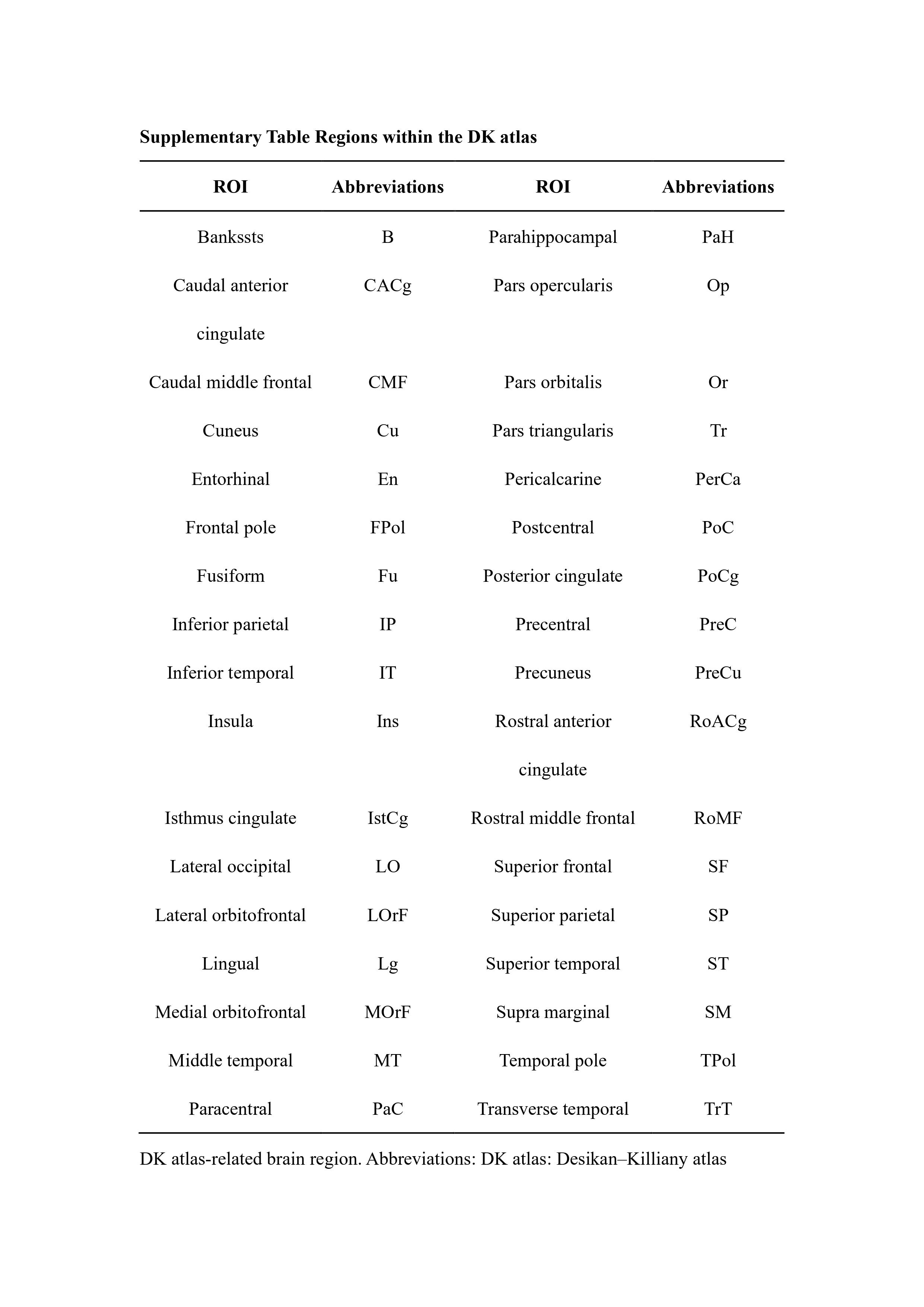

Supplement: Supplementary file 2 — Table S1. [file EPI4-9-368-s001.jpg]
